# The role of traditional Chinese medicine in modulating gut microbiota to alleviating insulin resistance in polycystic ovary syndrome

**DOI:** 10.3389/fnut.2025.1700612

**Published:** 2025-11-25

**Authors:** Lin Yin, Wanqiu Yang, Qingling Xie, Jili Xu, Ying Lan, Jie Wu

**Affiliations:** 1 Chengdu University of Traditional Chinese Medicine, Chengdu, China; 2 Hospital of Chengdu University of Traditional Chinese Medicine, Chengdu, China

**Keywords:** polycystic ovary syndrome, gut microbiota, insulin resistance, traditional Chinese medicine, natural products, short-chain fatty acid

## Abstract

Polycystic ovary syndrome (PCOS) is a common endocrine and metabolic disorder characterized by hyperandrogenism, anovulation, and insulin resistance (IR). Recent evidence suggests that gut microbiota (GM) dysbiosis contributes to PCOS pathophysiology, connecting metabolic, immune, and hormonal disturbances. Reduced microbial diversity, depletion of short-chain fatty acid (SCFA)-producing bacteria, and enrichment of endotoxin-producing taxa disrupt intestinal barrier integrity, promote low-grade inflammation, and aggravate IR, thereby fueling a vicious cycle of hyperinsulinemia and hyperandrogenism. Traditional Chinese medicine (TCM) has shown unique advantages in modulating GM and alleviating PCOS-IR. Herbal formulas, active compounds (e.g., berberine), acupuncture, and dietary therapies such as inulin, quinoa, and flaxseed oil restore microbial balance, enhance SCFA production, regulate bile acid metabolism, and strengthen gut barrier function. These effects mitigate endotoxemia, suppress chronic inflammation, and improve insulin sensitivity. This review summarizes advances in understanding the role of GM in PCOS-IR and emphasizes TCM as a promising microbiota-targeted therapeutic approach.

## 1 Introduction

PCOS is a common gynecological and endocrine disorder that involves both reproductive and metabolic dysfunctions. It affects approximately 5 to 20% of women of reproductive age worldwide and is recognized as a leading cause of anovulatory infertility (1, 2). The main characteristics of PCOS include ovulatory dysfunction, hyperandrogenism, and polycystic ovaries, and it is often accompanied by IR (3). IR is present in an estimated 50 to 70% of women with PCOS and is associated with a range of adverse outcomes (4, 5). In the short term, it increases the risk of obesity, gestational diabetes and miscarriage, while in the long term, it contributes to the development of hyperlipidemia, type 2 diabetes mellitus (T2DM), metabolic syndrome, and cardiovascular disease (6, 7). These complications not only threaten the physical and mental health of patients but also complicate clinical treatment. Therefore, a comprehensive understanding of the mechanisms underlying insulin resistance in PCOS is essential for developing effective treatment strategies and enhancing both metabolic and reproductive outcomes for women affected by PCOS.

Recent studies have highlighted the crucial role of GM in the development of IR and PCOS (8, 9). As a symbiotic microorganism colonized in the human intestine, GM plays an essential role in regulating the host’s metabolic, immune, and endocrine functions (10). Under healthy conditions, a dynamic balance exists between GM and the host, sustaining intestinal homeostasis. However, women with PCOS exhibit gut microbial dysbiosis, characterized by a decrease in beneficial probiotics and an increase in pathogens in the GM (11, 12). The imbalance of microbes undermines the integrity of the gut barrier, increasing gut permeability and allowing the bacterial endotoxin lipopolysaccharide (LPS) to enter the systemic circulation; the presence of LPS in the bloodstream activates the host’s immune response and pro-inflammatory signaling pathways, which interfere with insulin receptor function and promote the development of IR (13, 14). Additionally, GM and its metabolites influence metabolic regulation by stimulating the secretion of brain-gut peptides, promoting pancreatic β-cell proliferation, and reducing insulin sensitivity (15). These effects result in compensatory hyperinsulinemia, further exacerbating IR. Therefore, maintaining a healthy and balanced GM is essential for mitigating insulin resistance and associated metabolic disturbances in PCOS.

Globally, the treatment of PCOS mainly focuses on conventional therapies like oral contraceptives, insulin sensitizers, and ovulation-inducing agents. However, these options are often limited by side effects, costs, and long-term safety concerns. As a result, more patients with PCOS are turning to complementary and alternative medicine (CAM), especially traditional Chinese medicine (TCM) such as herbs, acupuncture, and dietary supplements (16). TCM, a major branch of CAM, has been practiced for thousands of years and is widely used in treating female reproductive disorders, including PCOS (10, 17). Chinese guideline for diagnosis and management of PCOS includes TCM as an auxiliary treatment method. Accumulating evidence suggests that certain individual herbs and herbal formulas containing multiple bioactive compounds have the potential to regulate menstruation, stimulate ovulation, reduce inflammation, and alleviate metabolic dysfunction (18–20). Importantly, they have been shown to exert their therapeutic effects possibly through modulating the GM (21). These oral herbal medicines interact directly with the GM, altering microbial composition and boosting the production of beneficial metabolites like short-chain fatty acids (SCFAs), which are crucial for maintaining metabolic balance and insulin sensitivity (22). Therefore, TCM offers a promising complementary approach to managing PCOS-related insulin resistance. This review explores the current understanding of how TCM ameliorate PCOS-IR through GM modulation, providing a novel perspective for integrative therapeutic strategies.

## 2 Interaction between PCOS and IR

### 2.1 The relationship of IR and HA

**Figure 1:**
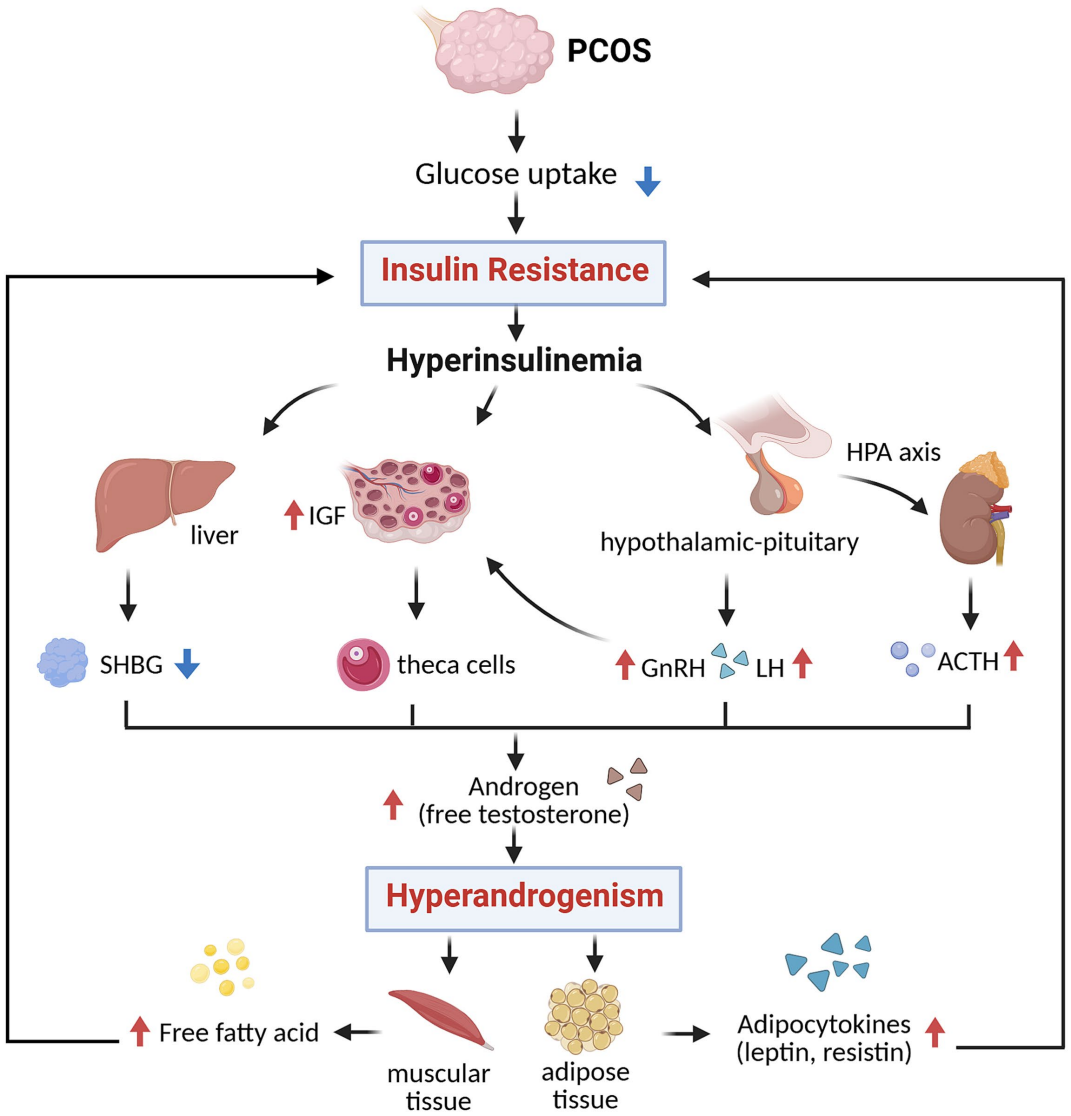
The relationship of IR and HA (created with biorender.com). SHBG, sex hormone-binding globulin; IGF, insulin-like growth factor; GnRH, gonadotropin-releasing hormone; LH, luteinizing hormone; ACTH, adrenocorticotropic hormone.

The interplay between IR and hyperandrogenism (HA) is central to the pathogenesis of PCOS, forming negative feedback that drives both metabolic and reproductive dysfunction. In women with PCOS, IR leads to impaired glucose uptake, resulting in compensatory hyperinsulinemia (HI), which stimulates androgen production by ovarian theca cells. It also suppresses hepatic synthesis of sex hormone-binding globulin (SHBG), thereby elevating circulating free testosterone levels. Additionally, HI promotes neuroendocrine disturbances by enhancing gonadotropin-releasing hormone (GnRH) expression and luteinizing hormone (LH) secretion, further increasing ovarian androgen production. Moreover, IR disrupts the hypothalamic–pituitary–adrenal (HPA) axis, increasing adrenocorticotropic hormone (ACTH) levels and adrenal androgen synthesis (23). These mechanisms converge to exacerbate HA, which in turn contributes to worsening IR by promoting visceral adiposity, reducing adiponectin and GLUT4 expression, and impairing insulin-stimulated glucose uptake in skeletal muscle (24). This pathological loop fosters a pro-inflammatory and lipotoxic state, characterized by enlarged, dysfunctional adipocytes and dysregulated adipokine secretion, marked by decreased insulin-sensitizing adiponectin and elevated levels of leptin, resistin, and chemerin. HI also directly alters ovarian granulosa cell function by prematurely upregulating LH receptors, leading to early differentiation, follicular arrest, and anovulation. Furthermore, hyperinsulinemia enhances cytochrome P450c17 activity and increases insulin-like growth factor-1 (IGF-1) bioavailability, further stimulating androgen biosynthesis (25). These disturbances are not limited to reproduction; they contribute to adverse pregnancy outcomes such as miscarriage and gestational diabetes, and promote the development of metabolic complications. Nevertheless, current evidence suggests that anti-IR treatment can decrease circulating levels of androgens and alleviate the phenotypes of PCOS (26) (Figure 1).

### 2.2 IR and metabolic dysfunction in PCOS

Compensatory hyperinsulinemia driven by IR not only promotes androgen excess but also leads to metabolic disturbances such as dysglycemia and dyslipidemia. Women with PCOS have approximately a threefold increased risk of developing impaired glucose tolerance (IGT), T2DM, and gestational diabetes mellitus (GDM) (27, 28). Lipid abnormalities are also prevalent in PCOS, with a characteristic profile of elevated triglycerides, reduced high-density lipoprotein (HDL) cholesterol, and increased levels of low-density lipoprotein (LDL) particles, changes that elevate cardiovascular risk (25). Furthermore, metabolic dysfunction-associated steatotic liver disease (MASLD) is increasingly recognized in PCOS and is driven by androgen excess, IR, and enhanced lipolysis, leading to hepatic fat accumulation (29). Accordingly, early intervention can alleviate the long-term health and economic burdens of patients with PCOS.

## 3 GM dysbiosis promotes PCOS-IR

### 3.1 GM and PCOS-IR

The GM is essential in preserving immune, metabolic, and endocrine homeostasis (15). The most dominant bacterial phyla in the gut include Firmicutes and Bacteroidetes, which constitute about 90% of the gut microbiome. Within Firmicutes, the genus *Clostridium* is highly dominant, while *Bacteroides* and *Prevotella* are the major representatives of Bacteroidetes (30). Other key phyla include Actinobacteria, mainly represented by *Bifidobacterium*, as well as smaller proportions of Proteobacteria, Fusobacteria, and Verrucomicrobia (31). Under physiological conditions, these microbiomes maintain intestinal barrier integrity, modulate systemic inflammation, and support metabolic homeostasis through their close communication with intestinal epithelial and immune cells. The disruption of this delicate balance, termed dysbiosis, which is characterized by reduced microbial diversity, an imbalance in microbial composition, and compromised gut barrier function, has been implicated in the pathogenesis of various metabolic disorders (15, 32, 33).

Recent evidence suggests that PCOS is possibly related to dysbiosis, one of the primary features of dysbiosis is the reduction of microbial richness and diversity. GM diversity is a critical marker of intestinal and systemic health, influencing metabolism, immune balance, and inflammation (34). It is commonly assessed using alpha (*α*) diversity, which reflects the richness and evenness of species within a sample, and beta (*β*) diversity, which measures compositional differences across individuals. In women with PCOS, several studies have reported a reduction in both *α*- and *β*-diversity, particularly in cases with IR (9, 35). In both clinical studies and animal models (e.g., letrozole-induced PCOS mice), decreased α- and β-diversity have been linked to altered gut microbial compositions, including a higher Firmicutes-to-Bacteroidetes ratio and increased abundance of pro-inflammatory gram-negative bacteria such as *Escherichia*, *Shigella*, and *Bacteroides* (11, 36). Inversely, some studies report no significant change in *α*-diversity in PCOS patients with normal BMI, while others suggest that observed changes are more closely tied to obesity or hyperandrogenism than to PCOS itself (37, 38). Factors such as diagnostic criteria, BMI, sex hormones, race, geography, eating habits, and host genetics may contribute to these discrepancies (38, 39). Overall, while dysbiosis is evident in PCOS, the extent of microbial diversity changes remains controversial.

Clinical studies consistently report alterations in microbial composition, characterized by the enrichment of pro-inflammatory and pathogenic taxa and depletion of beneficial commensals. A meta-analysis of 28 studies revealed a consistent enrichment of *Bacteroides*, *Parabacteroides*, *Fusobacterium*, and *Escherichia/Shigella*, and a reduction in *Lachnospira* and *Prevotella*, suggesting a shift toward a pro-inflammatory microbiome (11). Further evidence associated these microbial shifts with reduced gut–brain peptides (serotonin, ghrelin, and PYY), increased testosterone, and altered BMI (35). Elevated GABA-producing bacteria such as *Parabacteroides distasonis*, *Bacteroides fragilis*, and *Escherichia coli* were also found to correlate with elevated LH and LH/FSH ratios (40). In obese adolescent girls with PCOS, higher relative abundance of Actinobacteria and Streptococcaceae, along with reduced Bacteroidaceae, has been reported. Importantly, several studies have distinguished PCOS-IR from non-IR phenotypes (41). Significantly higher levels of *Rothia*, *Enterococcus*, *Ruminococcus*, and Bacteroidaceae, together with reduced Prevotellaceae, were observed in PCOS-IR patients, correlating with IR, inflammation, and hormonal disruption. Supporting these findings on the dysbiosis, animal models have confirmed causality (37, 42). Overgrowth of *Bacteroides vulgatus* induced IR and reproductive dysfunction in mice (3), while microbiota depletion reversed IR and enhanced Farnesoid X receptor (FXR) signaling in PCOS models (9). Other studies using letrozole- or DHEA-induced PCOS mice demonstrated increased Firmicutes and steroidogenic bacteria (e.g., Clostridiaceae, Nocardiaceae), alongside decreased beneficial taxa such as *Akkermansia*, *Turicibacter*, and *Clostridium sensu stricto* (36, 43, 44). Collectively, these findings underscore that gut microbial alterations in PCOS and PCOS-IR are closely linked to metabolic, endocrine, and inflammatory disruptions, reinforcing the GM as a potential therapeutic target (Table 1).

**Table 1:** Investigations on regulating GM composition in PCOS.

Investigation	Human/PCOS-like model	GM changes	Key Associations
Liang et al. (40)	Human (20 PCOS, 20 controls)	↑ *Parabacteroides distasonis*, *Bacteroides fragilis*, *E. coli*	Positively correlated with LH levels and LH: FSH ratios
Li et al. (11)	Meta-analysis (28 studies; 1,022 PCOS, 928 controls)	↓ *Lachnospira*, *Prevotella*; ↑ *Bacteroides*, *Parabacteroides*, *Lactobacillus*, *Fusobacterium*, *Escherichia/Shigella*	Indicative of pro-inflammatory dysbiosis
Liu et al. (10)	Human (33 PCOS, 15 controls)	↑ *Bacteroides*, *Escherichia/Shigella*, *Streptococcus*; ↓ *Akkermansia*, *Ruminococcaceae*	Altered with BMI, testosterone, and decreased ghrelin and PYY
Jobira et al. (41)	Obese adolescents (58 total)	↑ *Actinobacteria*, *Streptococcaceae*; ↓ *Bacteroidetes*, *Bacteroidaceae*, *Porphyromonadaceae*	PCOS-related changes in phylum/family levels in obese adolescents
Rodriguez Paris et al. (38)	DHT-induced PCOS-like mice	↓ *Bacteroides acidifaciens*	Inversely associated with obesity
Qi et al. (3)	Human and mice	↑ *Bacteroides vulgatus*	Promoted IR, altered bile acid metabolism, reduced IL-22, and induced infertility
Kelley et al. (36)	Letrozole-induced PCOS mice	↓ *Bacteroidetes*; ↑ *Firmicutes*	Time-dependent changes associated with metabolic disturbances
Sherman et al. (43)	PCOS-like rats	↑ *Nocardiaceae*, *Clostridiaceae*; ↓ *Akkermansia*, *Bacteroides*, *Lactobacillus*, *Clostridium*	Enriched for steroid hormone-related bacteria
Han et al. (44)	DHEA-induced PCOS-like rats	↓ *Turicibacter*, *Clostridium sensu stricto*	Associated with glucose metabolism and fiber response
He et al. (37)	Human (PCOS-IR, PCOS-NIR, controls)	↑ *Rothia*, *Ruminococcus*, *Enterococcus*; ↓ *Prevotella*	Linked to IR, blood pressure, waist/hip circumference
Zeng et al. (42)	Human (25 total)	↑ *Bacteroidaceae*; ↓ *Prevotellaceae*	Abundance correlated with IR, inflammation, and hormone levels
Yang et al. (9)	Human and mice	↑ *Bacteroides*	Gut microbiota removal reversed IR and upregulated FXR/FGF15 expression

### 3.2 The LPS and damaged gut barrier promote IR in PCOS

It is well known that the pathogenesis and development of PCOS is closely related to chronic low-grade inflammation, one of the key drivers of which is LPS, a pro-inflammatory endotoxin derived from Gram-negative gut bacteria (45, 46). The gut barrier is destroyed due to the GM dysbiosis in PCOS patients, allowing the transfer of LPS into systemic blood circulation and inducing metabolic endotoxemia (14). The dysbiosis characterized by an overgrowth of Gram-negative bacteria such as Bacteroidaceae, *Escherichia coli*, *Desulfovibrio*, and *Burkholderia* leads to increased LPS production in the gut (11, 47). Under normal conditions, tight junction proteins such as occludin and ZO-1 maintain the integrity of the intestinal mucosal barrier. However, dietary factors such as high saturated fat intake and low fiber consumption compromise barrier function, increase gut permeability, and allow LPS to translocate into the bloodstream, which may be an early factor in the development of inflammation and IR in humans and mice (48, 49). In PCOS, patients often exhibit decreased expression of occludin and ZO-1, resulting in a “leaky gut” and elevated circulating LPS levels (50, 51). Once in the bloodstream, LPS binds to LPS-binding protein (LBP) and is recognized by the CD14/Toll-like receptor 4 (TLR4) complex on immune cells and various tissues, including ovarian theca cells. This interaction activates MyD88-dependent signaling cascades, leading to nuclear factor-κB (NF-κB) activation and the release of pro-inflammatory cytokines such as TNF-*α*, IL-1β, and IL-6 (49, 52). These cytokines interfere with insulin receptor signaling by promoting suppressor of cytokine signaling-3 (SOCS-3) expression and serine phosphorylation of insulin receptor substrate-1 (IRS-1), ultimately impairing GLUT4-mediated glucose uptake and exacerbating IR (53). For instance, TNF-*α* has been shown to cause IR by increasing serine phosphorylation on IRS-1 (54). IL-6, notably elevated in PCOS, further inhibits insulin signaling and contributes to IR and ovarian dysfunction by disrupting follicular development (55). Experimental models have demonstrated that high-fat diets elevate LPS levels and induce IR (56). Notably, direct LPS injection elevates fasting glucose and insulin levels, confirming its pathogenic role (56). Emerging evidence suggests that modulating GM composition and restoring gut barrier integrity, such as through probiotics and Chinese herbal medicine, may reduce LPS translocation and inflammatory signaling. Collectively, in patients with PCOS, GM dysbiosis leads to elevated LPS levels, which increase intestinal permeability, impair insulin receptor function, and trigger a persistent inflammatory response, driving the progression of the PCOS-IR phenotype.

### 3.3 The products of GM promote IR

#### 3.3.1 Short-chain fatty acids (SCFAs)

SCFAs, predominantly acetate, propionate, butyrate, and valerate, are critical microbial metabolites produced through the fermentation of dietary fibers by GM, which plays a vital role in metabolic regulation (57). Studies show that women with PCOS have lower levels of SCFA-producing bacteria such as *Butyricimonas*, *Blautia*, *Coprococcus*, and *Faecalibacterium prausnitzii,* leading to decreased SCFA levels, especially butyrate, which may contribute to IR (11). SCFAs exert their effects via activation of G protein-coupled receptors (GPR41, GPR43, GPR109A) and free fatty acid receptors (FFAR2/3) expressed on intestinal epithelial cells, enteroendocrine cells, adipose tissue, and pancreatic β-cells (58). This signaling promotes the secretion of gut hormones such as glucagon-like peptide-1 (GLP-1) and peptide YY (PYY), which improve insulin sensitivity and regulate energy homeostasis (59). Clinical evidence indicates that women with PCOS exhibit lower fecal SCFA levels compared to controls, with reductions inversely associated with fasting insulin (60). Zhang et al. reported significantly higher fecal SCFAs in healthy women than in those with PCOS, while dietary or probiotic interventions restoring SCFA production improved glycemic and lipid profiles (61). Probiotic supplementation, including strains like *Bifidobacterium*, has been shown to restore SCFA production, improve GLP-1 secretion, and enhance glycemic control in PCOS patients (61). In mouse models, butyrate supplementation not only prevented obesity and IR on a high-fat diet but also enhanced mitochondrial function and energy expenditure (62). Collectively, reduced SCFA levels, especially butyrate, due to gut microbial dysbiosis appear to play a pivotal role in the development of IR in PCOS. Besides, SCFAs strengthen the intestinal barrier and reduce LPS-induced endotoxemia by inhibiting inflammatory pathways like NF-κB, suppressing pro-inflammatory cytokines (e.g., TNF-*α*, IL-1β), and increasing the expression of tight junction proteins (33). Collectively, reduced SCFA levels due to gut microbial dysbiosis contribute to the development of IR in PCOS.

#### 3.3.2 Bile acid (BAs)

BAs are not only critical for lipid digestion and absorption but also function as potent signaling molecules that regulate glucose and lipid metabolism, inflammation, and energy homeostasis (63). Primary BAs, synthesized in the liver as cholic acid and chenodeoxycholic acid, undergo microbial transformations in the intestine by bacteria such as *Lactobacillus*, *Bifidobacterium*, and *Bacteroides*, generating secondary BAs including deoxycholic acid (DCA), lithocholic acid (LCA), and ursodeoxycholic acid (UDCA) (63). In PCOS, gut microbiota dysbiosis disrupts this transformation, leading to reduced beneficial bile acids like glycodeoxycholic acid (GDCA) and tauroursodeoxycholic acid (TUDCA) (13). For instance, *Bacteroides vulgatus* overgrowth increases bile salt hydrolase (bsh) gene abundance, lowering GDCA and TUDCA levels and inducing insulin resistance and hormonal imbalance. Supplementation with GDCA or TUDCA can restore ovarian and metabolic function by activating the BA–IL-22 signaling axis (3). Besides, BAs enhance insulin sensitivity via two major receptors: FXR and Takeda G-protein receptor 5 (TGR5) (64). FXR activation suppresses gluconeogenesis and promotes glycogen synthesis through the PI3K/AKT pathway, while TGR5 activation in intestinal endocrine cells increases GLP-1 secretion and regulates appetite via the gut–brain axis (65). In PCOS, impaired FXR/TGR5 signaling due to dysbiosis contributes to IR, hyperlipidemia, and chronic low-grade inflammation.

### 3.4 Brain-gut peptides

Recent evidence suggests that the pathogenesis of PCOS involves the gut-brain axis, a bidirectional network connecting the gut microbiota, the enteric nervous system, and central neuroendocrine circuits. Brain-gut peptides such as GLP-1, PYY, serotonin, and ghrelin are important factors in this axis. A clinical report indicated that the levels of ghrelin and PYY in the plasma of PCOS patients are significantly reduced, which is associated with an increase in the abundance of *Bacteroides, E. coli/Shigella*, and a decrease in beneficial bacteria such as *Akkermansia* (35). Ghrelin not only regulates appetite and energy balance but also influences hypothalamic gonadotropin-releasing hormone GnRH secretion, modulating LH release and ovarian function. Similarly, PYY and GLP-1 promote satiety, delay gastric emptying, and enhance insulin sensitivity; their reduction exacerbates metabolic dysfunction and hyperinsulinemia. Studies have shown that fasting and postprandial GLP-1 levels are lower in both lean and obese PCOS women compared to healthy controls, and this deficiency persists despite oral contraceptive treatment (66). In addition, ghrelin/obestatin imbalance and leptin dysregulation have been associated with altered lipid intake and increased HOMA-IR, further linking dietary composition, microbiota, and hormonal imbalance (67). Moreover, gut microbes can modulate brain–gut peptides through the vagus nerve and neurotransmitter production, including serotonin and GABA, influencing both appetite control and hypothalamic–pituitary–ovarian (HPO) axis activity. Collectively, diminished gut–brain peptide signaling driven by microbial dysbiosis and altered metabolite production contributes to the bidirectional loop between metabolic and reproductive dysfunction in PCOS.

**Figure 2:**
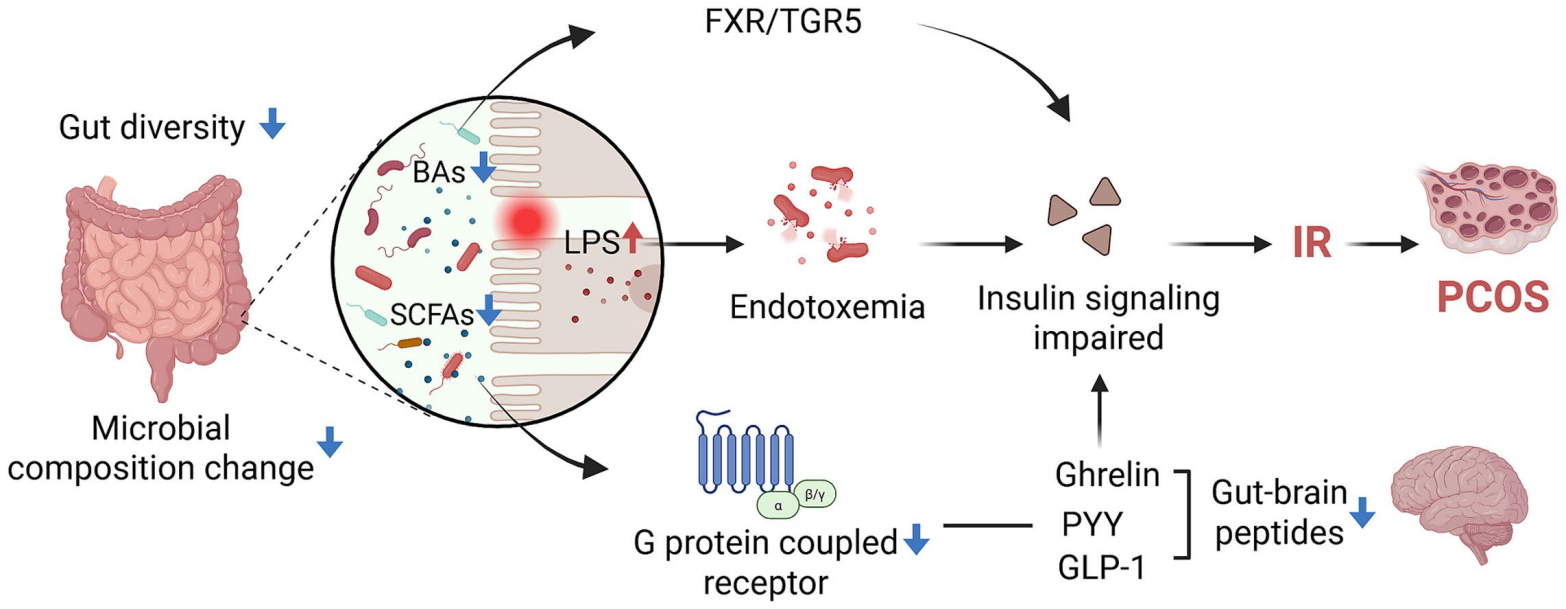
The mechanism of GM affects IR in PCOS patients (created with biorender.com).

In summary, gut dysbiosis promotes PCOS-related IR by disrupting intestinal barrier integrity, elevating LPS-induced inflammation, and altering microbial metabolites such as SCFAs and bile acids. These changes impair brain-gut axis signaling, reduce GLP-1 and PYY secretion, and disrupt hormone balance, forming a vicious cycle of metabolic inflammation and reproductive dysfunction in PCOS (Figure 2).

## 4 TCM as GM modulator in PCOS-IR

### 4.1 Herbal formulas

**Figure 3:**
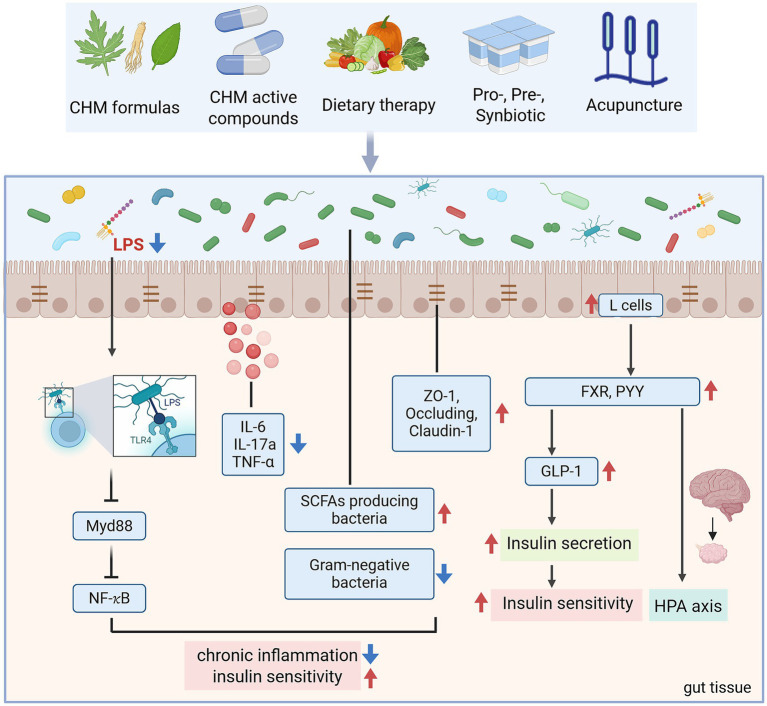
The mechanism of TCM alleviates PCOS-IR through GM (created with biorender.com).

Accumulating evidence highlights Chinese herbal medicine as a promising therapeutic method for PCOS, particularly by targeting GM to alleviate IR and chronic inflammation. Both clinical studies and PCOS-like animal models demonstrate that herbal formulas remodel gut microbial communities, restore intestinal barrier integrity, and regulate host metabolic signaling (Table 2; Figure 3). For instance, the Bu Shen Hua Zhuo Formula (BSHZF) reduced hyperandrogenism, fasting glucose, and IR in letrozole-induced PCOS rats while restoring microbial *α*-diversity, enriching *Lactobacillus* and SCFA-producing bacteria, and suppressing the TLR4/NF-κB inflammatory pathway by lowering serum LPS (21). Similarly, the Shaoyao-Gancao Decoction (SGD) alleviated hyperandrogenism, estrous cycle disruption, and ovarian inflammation by increasing beneficial bacteria such as *Akkermansia*, *Blautia*, and *Butyricicoccus*, reducing LPS-producing *Proteobacteria*, enhancing tight junction proteins, and inhibiting TLR4/NF-κB activation (68). Moreover, SGD was shown to regulate BA-related microbes and the BA/FXR pathway, suggesting dual actions on microbial and metabolic signaling (69).

**Table 2:** Effects of CHM Formulas and active compounds on GM and PCOS-IR.

CHM	Human/Model	GM changes	Signaling pathways	Metabolic/reproductive outcomes
Formulas				
Bu Shen Hua Zhuo Formula (BSHZF)	Letrozole-induced PCOS rats	↑ *Lactobacillus*, SCFA-producing bacteria (*Allobaculum*, *Bacteroides*, *Ruminococcaceae*), ↓ *Firmicutes*	↓ LPS, TLR4/NF-κB inhibition	↓ Body weight, fasting glucose, insulin, testosterone; improved ovarian morphology
Shaoyao-Gancao Decoction (SGD)	Letrozole-induced PCOS rats	↓ Firmicutes/Bacteroidetes ratio; ↓ *Proteobacteria*; ↑ *Butyricicoccus*, *Coprococcus*, *Akkermansia*, *Blautia*, *Bacteroides*	Enhanced tight junctions; ↓ TLR4/NF-κB	Improved estrous cycles, reduced hyperandrogenism, ↓ inflammation
SGD (extended)	PCOS rats (fecal transplant study)	Remodelled BA-related bacteria	BA/FXR pathway regulation	Ameliorated dyslipidemia, estrous dysfunction
Bailing Capsules (BL)	DHEA + high-fat diet PCOS mice	↑ *Akkermansia*, ↓ *Muribaculaceae*	IRS1/PI3K/AKT activation; ↓ TLR4/MyD88/NF-κB	Improved IR, hormone balance, ovarian morphology; ↓ inflammation
Heqi San (HQS)	DHEA + high-fat diet PCOS mice	↑ *Bifidobacterium*, *Parasutterella*; ↓ *Lachnoclostridium*	↓ NF-κB, M1 macrophage polarization; anti-apoptotic signaling	Improved IR, reduced granulosa apoptosis, ↓ IL-6, TNF-α, ovarian protection
Yulin Tong Bu Formula (YLTB)	DHEA + high-fat diet PCOS mice; pseudo-sterile models	Restored gut microbial diversity; metabolites (ferulic acid, folic acid) linked to PCOS parameters	Microbiota–metabolite interactions	Improved glucose clearance, insulin sensitivity, and ovarian function
Fufang Zhenzhu Tiao Zhi (FTZ)	Letrozole-induced PCOS mice	Not specified (systemic modulation)	↑ Adiponectin signaling	Restored estrous cycles, alleviated IR, improved ovarian morphology
Guizhi Fuling Wan (GFW)	Letrozole + high-fat diet PCOS rats	↑ *Alloprevotella*, ↓ pro-inflammatory *Lachnospiraceae*, *Ruminococcaceae*	↓ Inflammatory cytokines (TNF-α, IL-6, hs-CRP)	↓ Fasting insulin, improved IR and ovarian histology
Modified Banxia Xiexin Decoction	PCOS-IR rats (letrozole + high-fat diet)	↑ *Akkermansia*, *Blautia*, ↓ *Clostridium_sensu_stricto_1*	Barrier repair, metabolic regulation	Improved IR, corrected glucose metabolism
Modified Cangfu Daotan Decoction (MCDD)	PCOS-IR rats (letrozole + high-fat diet)	Not specified	↓ NF-κB/LCN-2; ↑ Insr/Irs-1/Glut4	↓ Body weight, restored estrous cycle, ↓ fatty liver, improved IR
Jiawei Qi Gong Wan	Clinical study in PCOS patients (phlegm-dampness syndrome)	↑ Butyrate-producing bacteria, ↓ LPS-producing and pro-inflammatory taxa	Microbiota–inflammation interaction	Improved IR, BMI, hormone balance, menstrual regulation
Active compounds				
Berberine (BBR)	Clinical trials in PCOS patients; DHEA-induced PCOS rats	↓ Firmicutes/Bacteroidetes ratio; modulated *Romboutsia, Bacteroides, Clostridium*	Regulated glucose and glutamine metabolism; modulated KEGG pathways (T2DM, ABC transporters); ↓ LPS	↓ Fasting insulin, HOMA-IR, testosterone; ↓ waist circumference; improved IR and metabolic profile
Naringenin (Nar)	Letrozole-induced PCOS rats	↑ *Butyricimonas, Lachnospira, Coprococcus, Roseburia*; ↓ *Prevotella*	↑ Tight junction proteins (claudin-1, occludin); modulated AMPK, SIRT1/PGC-1α	↓ Body weight; improved estrous cycles, ovarian morphology, IR, and hormone balance
Dendrobium officinale polysaccharide (DOP)	Letrozole-induced PCOS rats	↑ Butyrate-producing bacteria; ↑ α-diversity	↑ Butyrate and PYY; GPR41-mediated gut–brain–ovary axis	Improved ovarian morphology, estrous cycles, and endocrine function
Cordyceps polysaccharide (CP)	PCOS-like rats	↓ *Desulfovibrionaceae, Helicobacter* (Gram-negative bacteria)	↓ LPS translocation; inhibited TLR4/MyD88/NF-κB in liver and adipose; restored insulin signaling	↓ IR, improved glucose-lipid metabolism, alleviated ovarian polycystic lesions
Astragalus polysaccharide (APS)	DHEA-induced PCOS mice	↑ *Rikenellaceae, Odoribacter, Marinifilaceae*; ↓ *Bacteroidota*	Reduced oxidative stress; gut microbiota–metabolite correlations with glucose and lipids	↓ IR, OS, dyslipidemia; improved reproductive function
Mangiferin	Letrozole + HFD-induced PCOS rats	Altered Firmicutes, Bacteroidota, Proteobacteria; ↑ *Blautia, Coprococcus, Roseburia*	Anti-apoptotic (↓ Caspase-3, Cytc); modulated inflammation/apoptosis signaling	Improved lipid/glucose metabolism, IR, hormone balance, ovarian function
Curcumin	DHEA-induced PCOS mice	Not specified; improved barrier integrity	↑ Occludin, ZO-1; inhibited TLR4/MyD88/NF-κB; ↓ IL-6, TNF-α, LPS	↓ Body weight, testosterone, LH/FSH; ↑ insulin sensitivity, E2; improved ovarian/colon histology

Several classical prescriptions also show potential in regulating PCOS-IR through GM. Guizhi Fuling Wan (GZFL) improved IR and inflammation by reshaping microbial composition, notably restoring *Alloprevotella* and reducing inflammatory taxa (70). Modified Banxia Xiexin Decoction (BX) and Modified Cangfu Daotan Decoction (MCDD) attenuated hyperinsulinemia, reduced inflammatory cytokines, and modulated NF-κB and LCN-2 pathway (71, 72). Clinical evidence also supports these findings, Jiawei Qi Gong Wan (JQG) improved IR and endocrine dysfunction in PCOS patients with phlegm-dampness syndrome by increasing butyrate-producing bacteria, reducing LPS-producing species, and restoring microbial diversity (73).

Other formulas exhibit complementary benefits. Bailing capsules (BL) improved insulin sensitivity and ovarian function in DHEA-induced PCOS mice by repairing gut barrier integrity, reducing systemic inflammation, and inhibiting TLR4/NF-κB activation, while enriching *Akkermansia* (53). Heqi San (HQS) demonstrated anti-inflammatory effects by suppressing NF-κB activity, inhibiting macrophage M1 polarization, and preventing granulosa cell apoptosis, alongside enriching *Bifidobacterium* and *Parasutterella* (74). Yulin Tong Bu formula (YLTB) corrected ovarian dysfunction and glucose intolerance, with metabolomic analysis identifying ferulic acid as a key microbiota-associated mediator (75). Fufang Zhenzhu Tiao Zhi (FTZ) improved estrous cycle regularity and IR in letrozole-induced PCOS mice by upregulating adiponectin, supporting fat–ovary metabolic crosstalk (76). These findings underscore that CHM formulas act through enhancing SCFA-producing bacteria, modulating BA metabolism, reinforcing intestinal barrier function, suppressing LPS-induced inflammation, and restoring host metabolic pathways.

### 4.2 Bioactive compounds of herbal medicine

Modern studies have shown that bioactive compounds derived from herbal medicine play a therapeutic role in PCOS-IR by regulating the GM and its related metabolic pathways (Table 2; Figure 3). Berberine, an isoquinoline alkaloid derived from Coptis and Phellodendron species, has attracted considerable attention for its glucose- and lipid-lowering properties, with multiple studies supporting its potential to alleviate IR in PCOS. Clinical trials revealed that 12 weeks of berberine reduced waist circumference, HOMA-IR, and metabolic parameters more effectively than placebo and even metformin (77). Mechanistic studies suggest that berberine acts partly through reshaping the GM, enhancing SCFA producers, and regulating key metabolites such as glutamine and glucose, thereby influencing host energy metabolism and inflammatory pathways (78). However, the therapeutic role of berberine remains controversial. While certain PCOS-like rodent models confirmed improvements in IR, sex hormone profiles, and ovarian morphology, another study reported that berberine reduced microbial diversity without ameliorating metabolic or reproductive phenotypes (79). Such discrepancies may reflect differences in experimental models, treatment duration, or baseline microbiota composition.

Naringenin (Nar), a natural flavanone, has demonstrated substantial benefits in PCOS-like models. Nar restored estrous cycles, improved ovarian morphology, and attenuated hyperandrogenism while reducing body weight and improving IR. GM sequencing revealed Nar-induced enrichment of SCFA-producing bacteria (*Butyricimonas*, *Lachnospira*, *Coprococcus*, *Roseburia*), alongside upregulation of tight junction proteins (claudin-1, occludin) in the colon (80). These findings suggest that Nar exerts metabolic and reproductive improvements through enhancing intestinal barrier integrity and SCFA-driven signaling pathways.

Polysaccharides are another class of herbal medicine active ingredients with GM-dependent effects. Dendrobium officinale polysaccharide (DOP) cannot be directly absorbed but is fermented into SCFAs, particularly butyrate, by gut microbes. In PCOS rats, DOP increased microbial diversity, enriched butyrate producers, and elevated butyrate and PYY levels, which mediated improvements in ovarian morphology and estrous cyclicity via a gut–brain–ovary axis (81). Similarly, Cordyceps polysaccharide (CP) ameliorated glucose-lipid disturbances by reducing Gram-negative bacteria such as *Desulfovibrionaceae* and *Helicobacter*, thereby lowering gut-derived LPS translocation. This suppressed TLR4/MyD88/NF-κB activation in the liver and adipose tissue, restored insulin signaling, and alleviated ovarian polycystic changes (82). Astragalus polysaccharide (APS) also improved IR, oxidative stress, and dyslipidemia in PCOS mice, while reshaping GM by enriching beneficial bacteria such as *Odoribacter* and Marinifilaceae (83).

Other phytochemicals exhibit complementary effects. Mangiferin, a xanthone glycoside, ameliorated ovarian dysfunction, IR, and lipid abnormalities in PCOS rats while significantly altering gut microbial composition, increasing beneficial SCFA-producing genera (*Blautia, Coprococcus, Roseburia*). Transcriptomic analyses further suggested its regulation of apoptosis and inflammatory signaling (84). Curcumin, a well-known polyphenol, demonstrated anti-inflammatory and barrier-protective effects in PCOS models. It reduced serum testosterone and LH/FSH ratios, improved insulin sensitivity, and attenuated ovarian and colonic histopathology. Mechanistically, curcumin increased occludin and ZO-1 expression while suppressing TLR4/MyD88/NF-κB activation and systemic proinflammatory cytokines, thereby reducing LPS-induced endotoxemia (52). Another polyphenol, resveratrol, is widely used in the treatment of PCOS. Wang et al. showed that fecal microbiota transplantation (FMT) from resveratrol-treated donors significantly improved ovarian function and increased microbial diversity, characterized by elevated Firmicutes/Bacteroidetes ratios and higher relative abundance of *Lactobacillus murinus* and *L. salivarius* (85). Thus, GM as a central mediator of CHM active ingredients in alleviating PCOS-IR. By restoring gut microbial balance, reducing LPS leakage, and enhancing beneficial metabolites, these CHM bioactive compounds attenuate IR, hyperandrogenism, and ovarian dysfunction.

### 4.3 Dietary therapy

Dietary therapy, as a fundamental aspect of TCM, is increasingly being utilized in the research of PCOS (Figure 3). Recent experimental studies highlight the therapeutic role of functional foods such as quinoa and flaxseed oil (FO). In PCOS-like rats, quinoa supplementation significantly improved estrous cycle regularity, reduced fasting insulin and HOMA-IR, and alleviated ovarian, pancreatic, and intestinal pathology (86). Besides, quinoa restored autophagy and PI3K/AKT/mTOR signaling in ovarian tissue, reinforced intestinal barrier integrity via upregulation of tight junction proteins, and shifted GM composition by enriching *Lactobacillus*, *Bacteroides*, and *Oscillospira* while reducing *Prevotella* and the Firmicutes/Bacteroidetes ratio (86). These microbial and metabolic improvements were closely correlated with reductions in hyperandrogenism and improved reproductive outcomes. Similarly, flaxseed oil, rich in *α*-linolenic acid, exerted broad benefits in letrozole-induced PCOS rats (87). FO corrected sex hormone imbalances, reduced body weight and dyslipidemia, and ameliorated IR. Anti-inflammatory effects were evident through reductions in plasma and ovarian IL-1β, TNF-α, and MCP-1, alongside increases in IL-10. Importantly, FO supplementation enriched beneficial microbes including *Lactobacillus*, *Bifidobacterium*, and *Faecalibacterium*, while reducing Proteobacteria and Streptococcus (87).

Clinical evidence further supports the role of diet in PCOS pathophysiology. Meta-analyses reveal that women with PCOS consume significantly less dietary fiber than controls, a deficiency associated with greater adiposity, IR, and impaired glucose tolerance (88). Dietary fiber fermentation by gut microbes yields SCFAs, which regulate host metabolism, immune homeostasis, and gut barrier integrity. Inadequate fiber intake may reduce SCFA production, exacerbating PCOS metabolic disturbances (88). Integrating functional foods rich in fiber and unsaturated fatty acids may therefore represent a cost-effective, sustainable adjunct to conventional PCOS management.

### 4.4 Probiotics, prebiotics, and synbiotics

Prebiotics are organic substances that are not digested and absorbed by the host but can selectively promote the metabolism and proliferation of beneficial bacteria (22). Common prebiotics include cellulose, polysaccharides, chitosan, and polyphenols. Inulin, a fermentable dietary fiber that enhances SCFA production, improves microbial diversity, and mitigates systemic inflammation. Clinical trials have shown that inulin supplementation in PCOS women reduced body mass, hyperandrogenism, and IR while lowering inflammatory cytokines (TNF-*α*, IL-1β, IL-6, MCP-1) (89). Other studies in letrozole- or DHEA-induced PCOS mice confirmed that inulin increased SCFA production, restored estrous cycles, reduced testosterone, and suppressed ovarian inflammation via downregulation of LPS-TLR4 signaling (90, 91). Importantly, FMT from inulin-treated patients improved insulin sensitivity, lipid accumulation, and reproductive outcomes in antibiotic-treated mice (91).

Synbiotics, which combine probiotics and prebiotics, have demonstrated stronger effects than either alone. Usually comes as a supplement in pharmaceutical form of juice and capsules. In PCOS mouse models, inulin-enriched synbiotic yogurt restored estrous cyclicity, improved ovarian morphology, and enhanced IL-22 secretion while shifting microbial composition toward *Lactobacillus*, *Bifidobacterium*, and *Akkermansia*, with concurrent modulation of bile acid metabolism (92). Clinical studies further support these findings: randomized trials revealed that probiotic and synbiotic supplementation for 8–12 weeks significantly improved HOMA-IR, fasting glucose, lipid profiles, and hormonal balance (93). The meta-analysis confirmed that synbiotics exert the most pronounced improvements in metabolic and endocrine outcomes, though variations in probiotic strains, dosing, and trial designs limit standardization (93).

Probiotics are live microorganisms that confer health benefits by restoring microbial balance, enhancing gut barrier integrity, and modulating host immunity—particularly *Bifidobacterium* and *Lactobacillus* species. Specific bacterial species like *Bifidobacterium lactis* V9 supplementation in PCOS patients reduced LH/FSH ratios and increased SCFA levels, with clinical efficacy linked to successful gut colonization (61). In DHT-induced PCOS mice, *Bifidobacterium longum* BL21 supplementation enhanced ovarian function, improved glucose tolerance, and reduced inflammatory cytokines while enriching beneficial microbiota (94). Similarly, *Lactobacillus* strains alleviated hyperandrogenism, restored estrous cycles, and improved ovarian morphology in letrozole-induced PCOS models, highlighting the gut–brain–ovary axis as a potential regulatory pathway (95). Therefore, these interventions not only improve insulin sensitivity and metabolic health but also alleviate hyperandrogenism and ovarian dysfunction, highlighting their dual impact on both reproductive and metabolic outcomes. However, clinical evidence remains limited by small sample sizes and short intervention durations, necessitating larger multicenter trials to establish standardized protocols (Figure 3).

### 4.5 Acupuncture

Acupuncture, a cornerstone of TCM, has gained attention as a non-pharmacological intervention for PCOS and IR. In animal models, electroacupuncture (EA) improved estrous cyclicity, reduced visceral adiposity, and enhanced glucose tolerance in dihydrotestosterone (DHT)-induced PCOS rats. These benefits were associated with shifts in microbial taxa, notably reduced *Prevotella* and altered Tenericutes abundance (96). Human studies provide more interesting insights. A randomized trial combining acupuncture with clomiphene in obese PCOS patients demonstrated greater reductions in LH/FSH ratios and improved IR compared with clomiphene alone, alongside compositional changes in GM, including increased *Agathobacter faecis* and decreased *Erysipelatoclostridium* and *Streptococcus species*. These microbial shifts may contribute to improvements in hormone balance and metabolism (97). However, large-scale trials report mixed outcomes: Wen et al. found that acupuncture was less effective than metformin in reducing HOMA-IR, though it showed advantages in glucose metabolism and fewer gastrointestinal side effects. Such findings demonstrate its potential as a low-risk adjunct therapy, especially in patients intolerant to pharmacologic agents (98) (Figure 3).

## 5 Shortcomings and future prospection

Although TCMs show considerable promise as modulators of the GM in alleviating IR in PCOS, current evidence is limited by several shortcomings that warrant critical attention. Most clinical studies are small, single-center trials with short intervention durations and heterogeneous diagnostic criteria, making it difficult to generalize findings or establish standardized treatment regimens. While some clinical trials, such as those investigating berberine, demonstrate significant improvements in IR and metabolic parameters, contradictory findings in animal models highlight the complexity of herbal medicine–microbiota–host interactions and the need for greater mechanistic clarity. Moreover, the lack of long-term safety evaluations and rigorous quality control in herbal preparation, standardization, and bioactive compound identification poses significant challenges to reproducibility and clinical translation. Variations in formulation, dosage, and preparation methods further complicate the interpretation of therapeutic outcomes and hinder cross-study comparisons. Furthermore, most existing studies examine single herbs or isolated compounds, whereas traditional Chinese medicine typically employs multi-herb prescriptions with synergistic interactions that remain poorly characterized. Future research should integrate multi-omics technologies and artificial intelligence (AI)-driven analytical models to identify active components, predict host–microbiota interactions, and optimize individualized therapeutic strategies, and conduct well-designed, large-scale, multicenter randomized clinical trials with standardized diagnostic criteria and safety assessments to make TCM a safe, effective, and evidence-based strategy for managing PCOS and its metabolic dysfunctions.

## 6 Conclusion

PCOS is a multifactorial disorder in which IR and hyperandrogenism form a vicious cycle driving metabolic and reproductive dysfunction. Increasing evidence indicate that GM dysbiosis as a pivotal mediator of these abnormalities through mechanisms involving impaired intestinal barrier integrity, endotoxemia, disrupted microbial metabolites such as SCFAs and BAs, and altered gut–brain–ovarian signaling. Within this context, TCM emerges as a promising modulator of GM, capable of restoring microbial balance, reducing inflammation, and improving IR. Preclinical and clinical studies have shown that herbal formulas, active ingredients, dietary fibers, synbiotic interventions, and acupuncture enhance the abundance of SCFA-producing bacteria, strengthen intestinal barrier function, and attenuate systemic and ovarian inflammation. Moreover, these interventions often exert synergistic effects on metabolic and endocrine pathways, linking microbiota regulation to improved reproductive outcomes. Here, we emphasize that TCM may offer an integrative therapeutic strategy to alleviate IR and improve long-term outcomes in PCOS by regulating the gut microbiome.

## References

[1] Cunha A Póvoa AM. Infertility management in women with polycystic ovary syndrome: a review. Porto Biomed J. (2021) 6:e116. doi: 10.1097/j.pbj.0000000000000116, PMID: 33532657 PMC7846416

[2] Siddiqui S Mateen S Ahmad R Moin S. A brief insight into the etiology, genetics, and immunology of polycystic ovarian syndrome (PCOS). J Assist Reprod Genet. (2022) 39:2439–73. doi: 10.1007/s10815-022-02625-7, PMID: 36190593 PMC9723082

[3] Qi X Yun C Sun L Xia J Wu Q Wang Y . Gut microbiota–bile acid–interleukin-22 axis orchestrates polycystic ovary syndrome. Nat Med. (2019) 25:1225–33. doi: 10.1038/s41591-019-0509-0, PMID: 31332392 PMC7376369

[4] Stepto NK Cassar S Joham AE Hutchison SK Harrison CL Goldstein RF . Women with polycystic ovary syndrome have intrinsic insulin resistance on euglycaemic-hyperinsulaemic clamp. Hum Reprod. (2013) 28:777–84. doi: 10.1093/humrep/des463, PMID: 23315061

[5] El Leithy AA Abozaid M Al-Karmalawy AA Mahmoud Allam R Nourelden AZ Amer RM . Spirulina versus metformin for controlling some insulin signaling pathway genes in induced polycystic ovary syndrome rat model. Gene. (2024) 921:921. doi: 10.1016/j.gene.2024.148524, PMID: 38735598

[6] Escobar-Morreale HF. Polycystic ovary syndrome: definition, aetiology, diagnosis and treatment. Nat Rev Endocrinol. (2018) 14:270–84. doi: 10.1038/nrendo.2018.24, PMID: 29569621

[7] Ali AT. Polycystic ovary syndrome and metabolic syndrome. Cesk Gynekol. (2015) 80:279–89. PMID: 26265416

[8] Giampaolino P Foreste V Di Filippo C Gallo A Mercorio A Serafino P . Microbiome and PCOS: state-of-art and future aspects. Int J Mol Sci. (2021) 22:2048–63. doi: 10.3390/ijms22042048, PMID: 33669557 PMC7922491

[9] Yang Y-L Zhou W-W Wu S Tang W-L Wang Z-W Zhou Z-Y . Intestinal Flora is a key factor in insulin resistance and contributes to the development of polycystic ovary syndrome. Endocrinology. (2021) 10:1–16. doi: 10.1210/endocr/bqab118, PMID: 34145455 PMC8375444

[10] Liu M Yan J Wu Y Zhu H Huang Y Wu K. The impact of herbal medicine in regulating intestinal flora on female reproductive disorders. Front Pharmacol. (2022) 13:13. doi: 10.3389/fphar.2022.1026141, PMID: 36313343 PMC9614049

[11] Li P Shuai P Shen S Zheng H Sun P Zhang R . Perturbations in gut microbiota composition in patients with polycystic ovary syndrome: a systematic review and meta-analysis. BMC Med. (2023) 21:302. doi: 10.1186/s12916-023-02975-8, PMID: 37559119 PMC10413517

[12] Chu W Han Q Xu J Wang J Sun Y Li W . Metagenomic analysis identified microbiome alterations and pathological association between intestinal microbiota and polycystic ovary syndrome. Fertil Steril. (2020) 113:1286–98.e4. doi: 10.1016/j.fertnstert.2020.01.027, PMID: 32482258

[13] He F-f Li Y-m. Role of gut microbiota in the development of insulin resistance and the mechanism underlying polycystic ovary syndrome: a review. J Ovarian Res. (2020) 13:73. doi: 10.1186/s13048-020-00670-3, PMID: 32552864 PMC7301991

[14] Tremellen K Pearce K. Dysbiosis of gut microbiota (DOGMA) – a novel theory for the development of polycystic ovarian syndrome. Med Hypotheses. (2012) 79:104–12. doi: 10.1016/j.mehy.2012.04.016, PMID: 22543078

[15] Qi X Yun C Pang Y Qiao J. The impact of the gut microbiota on the reproductive and metabolic endocrine system. Gut Microbes. (2021) 13:1–21. doi: 10.1080/19490976.2021.1894070, PMID: 33722164 PMC7971312

[16] Shen W Jin B Pan Y Han Y You T Zhang Z . The effects of traditional Chinese medicine-associated complementary and alternative medicine on women with polycystic ovary syndrome. Evid Based Complement Alternat Med. (2021) 2021:1–26. doi: 10.1155/2021/6619597, PMID: 33727940 PMC7935578

[17] Jiang L Fei H Tong J Zhou J Zhu J Jin X . Hormone replacement therapy reverses gut microbiome and serum metabolome alterations in premature ovarian insufficiency. Front Endocrinol. (2021) 12:12. doi: 10.3389/fendo.2021.794496, PMID: 35002971 PMC8733385

[18] Liao W-T Su C-C Lee M-T Li C-J Lin C-L Chiang J-H . Integrative Chinese herbal medicine therapy reduced the risk of type 2 diabetes mellitus in patients with polycystic ovary syndrome: a nationwide matched cohort study. J Ethnopharmacol. (2019) 243:112091. doi: 10.1016/j.jep.2019.112091, PMID: 31325604

[19] Lai L Flower A Prescott P Wing T Moore M Lewith G. Standardised versus individualised multiherb Chinese herbal medicine for oligomenorrhoea and amenorrhoea in polycystic ovary syndrome: a randomised feasibility and pilot study in the UK. BMJ Open. (2017) 7:e011709. doi: 10.1136/bmjopen-2016-011709, PMID: 28159846 PMC5293993

[20] Dai M Shi B Zhang X Gu Y Wang F Zhou J . Effects of Chinese herbal medicine on pregnancy outcomes of women with PCOS undergoing in vitro fertilization and embryo transfer: a retrospective cohort study. Eur J Integr Med. (2024) 65:102317. doi: 10.1016/j.eujim.2023.102317

[21] Wang Y Xiao H Liu Y Tong Q Yu Y Qi B . Effects of Bu Shen Hua Zhuo formula on the LPS/TLR4 pathway and gut microbiota in rats with letrozole-induced polycystic ovary syndrome. Front Endocrinol. (2022) 13:13. doi: 10.3389/fendo.2022.891297, PMID: 36017323 PMC9396283

[22] Yin L XW-r Huang G-x Wendy Hsiao WL. Research progress on correlation between traditional Chinese medicine-gut microbiota and host’s own metabolic immune homeostasis. Chin Tradit Herb Drug. (2022) 53:2526–38. doi: 10.7501/j.issn.0253-2670.2022.08

[23] Zhao H Zhang J Cheng X Nie X He B. Insulin resistance in polycystic ovary syndrome across various tissues: an updated review of pathogenesis, evaluation, and treatment. J Ovarian Res. (2023) 16:9. doi: 10.1186/s13048-022-01091-0, PMID: 36631836 PMC9832677

[24] Bril F Ezeh U Amiri M Hatoum S Pace L Chen Y-H . Adipose tissue dysfunction in polycystic ovary syndrome. J Clin Endocrinol Metab. (2024) 109:10–24. doi: 10.1210/clinem/dgad356, PMID: 37329216 PMC10735305

[25] Helvaci N Yildiz BO. Polycystic ovary syndrome as a metabolic disease. Nat Rev Endocrinol. (2024) 21:230–44. doi: 10.1038/s41574-024-01057-w, PMID: 39609634

[26] Teede HJ Tay CT Laven J Dokras A Moran L Piltonen T . International Evidence-Based-Guidelines for the assessment and management of PCOS-2023. Monash University (2023). doi: 10.26180/24003834.v1

[27] Kakoly NS Khomami MB Joham AE Cooray SD Misso ML Norman RJ . Ethnicity, obesity and the prevalence of impaired glucose tolerance and type 2 diabetes in PCOS: a systematic review and meta-regression. Hum Reprod Update. (2018) 24:455–67. doi: 10.1093/humupd/dmy007, PMID: 29590375

[28] Sha T Wang X Cheng W Yan Y. A meta-analysis of pregnancy-related outcomes and complications in women with polycystic ovary syndrome undergoing IVF. Reprod Biomed Online. (2019) 39:281–93. doi: 10.1016/j.rbmo.2019.03.203, PMID: 31255606

[29] Kelley CE Brown AJ Diehl AM Setji TL. Review of nonalcoholic fatty liver disease in women with polycystic ovary syndrome. World J Gastroenterol. (2014) 20:14172–84. doi: 10.3748/wjg.v20.i39.14172, PMID: 25339805 PMC4202347

[30] Sangappa B Chadchan VS Ramakrishna Kommagani. Female reproductive dysfunctions and the gut microbiota. J Mol Endocrinol. (2022) 69:R81–94. doi: 10.1530/JME-21-0238 35900833 PMC10031513

[31] Rinninella E Raoul P Cintoni M Franceschi F Miggiano G Gasbarrini A . What is the healthy gut microbiota composition? A changing ecosystem across age, environment, diet, and diseases. Microorganisms. (2019) 7:14. doi: 10.3390/microorganisms7010014, PMID: 30634578 PMC6351938

[32] Zhang M Hu R Huang Y Zhou F Li F Liu Z . Present and future: Crosstalks between polycystic ovary syndrome and gut metabolites relating to gut microbiota. Front Endocrinol. (2022) 13:13. doi: 10.3389/fendo.2022.933110, PMID: 35928893 PMC9343597

[33] Bock PM Martins AF Schaan BD. Understanding how pre- and probiotics affect the gut microbiome and metabolic health. Amer J Physiol Endocrinol Metabol. (2024) 327:E89–E102. doi: 10.1152/ajpendo.00054.2024, PMID: 38809510

[34] Wastyk HC Fragiadakis GK Perelman D Dahan D Merrill BD Yu FB . Gut-microbiota-targeted diets modulate human immune status. Cell. (2021) 184:4137–53.e14. doi: 10.1016/j.cell.2021.06.019, PMID: 34256014 PMC9020749

[35] Liu R Zhang C Shi Y Zhang F Li L Wang X . Dysbiosis of gut microbiota associated with clinical parameters in polycystic ovary syndrome. Front Microbiol. (2017) 8:8. doi: 10.3389/fmicb.2017.00324, PMID: 28293234 PMC5328957

[36] Kelley ST Skarra DV Rivera AJ Thackray VG. The gut microbiome is altered in a Letrozole-induced mouse model of polycystic ovary syndrome. PLoS One. (2016) 11:e0146509. doi: 10.1371/journal.pone.0146509, PMID: 26731268 PMC4701222

[37] He F Li Y. The gut microbial composition in polycystic ovary syndrome with insulin resistance: findings from a normal-weight population. J Ovarian Res. (2021) 14:50. doi: 10.1186/s13048-021-00799-9, PMID: 33773586 PMC8005233

[38] Rodriguez Paris V Wong XYD Solon-Biet SM Edwards MC Aflatounian A Gilchrist RB . The interplay between PCOS pathology and diet on gut microbiota in a mouse model. Gut Microbes. (2022) 14:e2085961. doi: 10.1080/19490976.2022.2085961, PMID: 35787106 PMC9450977

[39] Vink JM Sadrzadeh S Lambalk CB Boomsma DI. Heritability of polycystic ovary syndrome in a Dutch twin-family study. J Clin Endocrinol Metab. (2006) 91:2100–4. doi: 10.1210/jc.2005-1494, PMID: 16219714

[40] Liang Z Di N Li L Yang D. Gut microbiota alterations reveal potential gut–brain axis changes in polycystic ovary syndrome. J Endocrinol Investig. (2021) 44:1727–37. doi: 10.1007/s40618-020-01481-5, PMID: 33387350

[41] Jobira B Frank DN Pyle L Silveira LJ Kelsey MM Garcia-Reyes Y . Obese adolescents with PCOS have altered biodiversity and relative abundance in gastrointestinal microbiota. J Clin Endocrinol Metabol. (2020) 105:e2134–44. doi: 10.1210/clinem/dgz263, PMID: 31970418 PMC7147870

[42] Zeng B Lai Z Sun L Zhang Z Yang J Li Z . Structural and functional profiles of the gut microbial community in polycystic ovary syndrome with insulin resistance (IR-PCOS): a pilot study. Res Microbiol. (2019) 170:43–52. doi: 10.1016/j.resmic.2018.09.002, PMID: 30292647

[43] Sherman SB Sarsour N Salehi M Schroering A Mell B Joe B . Prenatal androgen exposure causes hypertension and gut microbiota dysbiosis. Gut Microbes. (2018) 9:1–22. doi: 10.1080/19490976.2018.1441664, PMID: 29469650 PMC6219642

[44] Han Q Wang J Li W Chen Z-J Du Y. Androgen-induced gut dysbiosis disrupts glucolipid metabolism and endocrinal functions in polycystic ovary syndrome. Microbiome. (2021) 9:101. doi: 10.1186/s40168-021-01046-5, PMID: 33957990 PMC8103748

[45] Page MJ Kell DB Pretorius E. The role of lipopolysaccharide-induced cell Signalling in chronic inflammation. Chronic stress (Thousand Oaks, Calif). (2022) 6:24705470221076390. doi: 10.1177/24705470221076390, PMID: 35155966 PMC8829728

[46] Stephens M von der Weid PY. Lipopolysaccharides modulate intestinal epithelial permeability and inflammation in a species-specific manner. Gut Microbes. (2020) 11:421–32. doi: 10.1080/19490976.2019.1629235, PMID: 31203717 PMC7524286

[47] Senthilkumar H Arumugam M. Gut microbiota: a hidden player in polycystic ovary syndrome. J Transl Med. (2025) 23:443. doi: 10.1186/s12967-025-06315-7, PMID: 40234859 PMC11998441

[48] González F Considine RV Abdelhadi OA Acton AJ. Saturated fat ingestion promotes lipopolysaccharide-mediated inflammation and insulin resistance in polycystic ovary syndrome. J Clin Endocrinol Metabol. (2019) 104:934–46. doi: 10.1210/jc.2018-01143, PMID: 30590569 PMC6364509

[49] Saad MJA Santos A Prada PO. Linking gut microbiota and inflammation to obesity and insulin resistance. Physiology. (2016) 31:283–93. doi: 10.1152/physiol.00041.2015, PMID: 27252163

[50] Rizk FH El Saadany AA Elshamy AM Abd Ellatif RA El-Guindy DM Helal DS . Ameliorating effects of adropin on letrozole-induced polycystic ovary syndrome via regulating steroidogenesis and the microbiota inflammatory axis in rats. J Physiol. (2024) 602:3621–39. doi: 10.1113/JP285793, PMID: 38980987

[51] Banaszewska B Siakowska M Chudzicka-Strugala I Chang RJ Pawelczyk L Zwozdziak B . Elevation of markers of endotoxemia in women with polycystic ovary syndrome. Hum Reprod. (2020) 35:2303–11. doi: 10.1093/humrep/deaa194, PMID: 32869098

[52] Yang Q Wan Q Wang Z. Curcumin mitigates polycystic ovary syndrome in mice by suppressing TLR4/MyD88/NF-κB signaling pathway activation and reducing intestinal mucosal permeability. Sci Rep. (2024) 14:29848. doi: 10.1038/s41598-024-81034-5, PMID: 39617843 PMC11609283

[53] Guan H-r Li B Zhang Z-h Wu H-s Wang N Chen X-f . Exploring the efficacy and mechanism of bailing capsule to improve polycystic ovary syndrome in mice based on intestinal-derived LPS-TLR4 pathway. J Ethnopharmacol. (2024) 331:118274. doi: 10.1016/j.jep.2024.118274, PMID: 38697410

[54] Hotamisligil GS Peraldi P Budavari A Ellis R White MF Spiegelman BM. IRS-1-mediated inhibition of insulin receptor tyrosine kinase activity in TNF-α- and obesity-induced insulin resistance. Science. (1996) 271:665–70. doi: 10.1126/science.271.5249.665, PMID: 8571133

[55] Fulghesu AM Sanna F Uda S Magnini R Portoghese E Batetta B. Il-6 serum levels and production is related to an altered immune response in polycystic ovary syndrome girls with insulin resistance. Mediat Inflamm. (2011) 2011:1–8. doi: 10.1155/2011/389317, PMID: 21547256 PMC3086286

[56] Cani PD Amar J Iglesias MA Poggi M Knauf C Bastelica D . Metabolic endotoxemia initiates obesity and insulin resistance. Diabetes. (2007) 56:1761–72. doi: 10.2337/db06-1491 17456850

[57] Koh A De Vadder F Kovatcheva-Datchary P Bäckhed F. From dietary Fiber to host physiology: short-chain fatty acids as key bacterial metabolites. Cell. (2016) 165:1332–45. doi: 10.1016/j.cell.2016.05.041, PMID: 27259147

[58] Li J Qiao J Li Y Qin G Xu Y Lao K . Metabolic disorders in polycystic ovary syndrome: from gut microbiota biodiversity to clinical intervention. Front Endocrinol. (2025) 16:1526468. doi: 10.3389/fendo.2025.1526468, PMID: 40357203 PMC12066289

[59] Salehi S Allahverdy J Pourjafar H Sarabandi K Jafari SM. Gut microbiota and polycystic ovary syndrome (PCOS): understanding the pathogenesis and the role of probiotics as a therapeutic strategy. Probiotics Antimicrobial Proteins. (2024) 16:1553–65. doi: 10.1007/s12602-024-10223-5, PMID: 38421576

[60] Kukaev E Kirillova E Tokareva A Rimskaya E Starodubtseva N Chernukha G . Impact of gut microbiota and SCFAs in the pathogenesis of PCOS and the effect of metformin therapy. Int J Mol Sci. (2024) 25:10636. doi: 10.3390/ijms251910636, PMID: 39408965 PMC11477200

[61] Zhang J Sun Z Jiang S Bai X Ma C Peng Q . Probiotic *Bifidobacterium lactis* V9 regulates the secretion of sex hormones in polycystic ovary syndrome patients through the gut-brain axis. mSystems. (2019) 4:e00017–19. doi: 10.1128/msystems.00017-19, PMID: 31020040 PMC6469956

[62] Gao Z Yin J Zhang J Ward RE Martin RJ Lefevre M . Butyrate improves insulin sensitivity and increases energy expenditure in mice. Diabetes. (2009) 58:1509–17. doi: 10.2337/db08-1637, PMID: 19366864 PMC2699871

[63] Collins SL Stine JG Bisanz JE Okafor CD Patterson AD. Bile acids and the gut microbiota: metabolic interactions and impacts on disease. Nat Rev Microbiol. (2022) 21:236–47. doi: 10.1038/s41579-022-00805-x, PMID: 36253479 PMC12536349

[64] Mei Y Li W Wang B Chen Z Wu X Lin Y . Gut microbiota: an emerging target connecting polycystic ovarian syndrome and insulin resistance. Front Cell Infect Microbiol. (2025) 15:1508893. doi: 10.3389/fcimb.2025.1508893, PMID: 40134784 PMC11933006

[65] Cai J Rimal B Jiang C Chiang JYL Patterson AD. Bile acid metabolism and signaling, the microbiota, and metabolic disease. Pharmacol Ther. (2022) 237:108238. doi: 10.1016/j.pharmthera.2022.108238, PMID: 35792223

[66] Aydin K Arusoglu G Koksal G Cinar N Yazgan Aksoy D Yildiz BO. Fasting and post‐prandial glucagon like peptide 1 and oral contraception in polycystic ovary syndrome. Clin Endocrinol. (2014) 81:588–92. doi: 10.1111/cen.12468, PMID: 24730585

[67] Wu W Fan X Yu Y Wang Z Wang Y. Alteration of ghrelin/obestatin ratio in adolescence with polycystic ovarian syndrome. Gynecol Endocrinol. (2018) 34:36–9. doi: 10.1080/09513590.2017.1336216, PMID: 28649890

[68] Chang Z-p Deng G-f Shao Y-y Xu D Zhao Y-n Sun Y-f . Shaoyao-gancao decoction ameliorates the inflammation state in polycystic ovary syndrome rats via remodeling gut microbiota and suppressing the TLR4/NF-κB pathway. Front Pharmacol. (2021) 12:670054. doi: 10.3389/fphar.2021.670054, PMID: 34054541 PMC8155368

[69] Duan J-Q Sun Y-F Wang X Liu H-Y Chang Z-P Shao Y-Y . Shaoyao-Gancao decoction improves dyslipidemia in rats with polycystic ovary syndrome by reshaping the gut microbiota and regulating the bile acid/FXR pathway. J Asian Nat Prod Res. (2025) 25:1–14. doi: 10.1080/10286020.2025.2482072, PMID: 40131972

[70] Zhu Y Li Y Liu M Hu X Zhu H. Guizhi fuling wan, Chinese herbal medicine, ameliorates insulin sensitivity in PCOS model rats with insulin resistance via remodeling intestinal homeostasis. Front Endocrinol. (2020) 11:575. doi: 10.3389/fendo.2020.00575, PMID: 32973686 PMC7482315

[71] Zhao H Chen R Zheng D Xiong F Jia F Liu J . Modified banxia xiexin decoction ameliorates polycystic ovarian syndrome with insulin resistance by regulating intestinal microbiota. Front Cell Infect Microbiol. (2022) 12:854796. doi: 10.3389/fcimb.2022.854796, PMID: 35619648 PMC9127304

[72] Liu S Zhang Y Yang F Gu J Zhang R Kuang Y . Modified Cangfu Daotan decoction ameliorates polycystic ovary syndrome with insulin resistance via NF-κB/LCN-2 signaling pathway in inflammatory microenvironment. Front Endocrinol. (2022) 13:13. doi: 10.3389/fendo.2022.975724, PMID: 36440213 PMC9686851

[73] Zhang N Li C Guo Y Wu H-c Mannucci C. Study on the intervention effect of Qi gong Wan prescription on patients with phlegm-dampness syndrome of polycystic ovary syndrome based on intestinal flora. Evid Based Complement Alternat Med. (2020) 2020:6389034. doi: 10.1155/2020/6389034 33062017 PMC7545460

[74] Li J Liu D Zhao H Zhang P Cai F Li H . Chinese medicine compound prescription HeQi san ameliorates chronic inflammatory states and modulates gut flora in dehydroepiandrosterone-induced polycystic ovary syndrome mouse model. Int Immunopharmacol. (2024) 137:112491. doi: 10.1016/j.intimp.2024.112491, PMID: 38909499

[75] Su Y-N Wang M-J Yang J-P Wu X-L Xia M Bao M-H . Effects of Yulin Tong Bu formula on modulating gut microbiota and fecal metabolite interactions in mice with polycystic ovary syndrome. Front Endocrinol. (2023) 14:14. doi: 10.3389/fendo.2023.1122709, PMID: 36814581 PMC9939769

[76] Xu Y Tang J Guo Q Xu Y Yan K Wu L . Traditional Chinese medicine formula FTZ protects against polycystic ovary syndrome through modulating adiponectin-mediated fat-ovary crosstalk in mice. J Ethnopharmacol. (2021) 268:113587. doi: 10.1016/j.jep.2020.113587, PMID: 33212180

[77] Wei W Zhao H Wang A Sui M Liang K Deng H . A clinical study on the short-term effect of berberine in comparison to metformin on the metabolic characteristics of women with polycystic ovary syndrome. Eur J Endocrinol. (2012) 166:99–105. doi: 10.1530/eje-11-0616, PMID: 22019891

[78] Shen H-R Xu X Ye D Li X-L. Berberine improves the symptoms of DHEA-induced PCOS rats by regulating gut microbiotas and metabolites. Gynecol Obstet Investig. (2021) 86:388–97. doi: 10.1159/000518040, PMID: 34515131

[79] Zhang F Ma T Cui P Tamadon A He S Huo C . Diversity of the gut microbiota in Dihydrotestosterone-induced PCOS rats and the pharmacologic effects of Diane-35, probiotics, and Berberine. Front Microbiol. (2019) 10:10. doi: 10.3389/fmicb.2019.00175, PMID: 30800111 PMC6375883

[80] Wu Y-X Yang X-Y Han B-S Hu Y-Y An T Lv B-H . Naringenin regulates gut microbiota and SIRT1/ PGC-1ɑ signaling pathway in rats with letrozole-induced polycystic ovary syndrome. Biomed Pharmacother. (2022) 153:113286. doi: 10.1016/j.biopha.2022.113286, PMID: 35724506

[81] Feng X Wang D Hu L Lu H ling B Huang Y . *Dendrobium officinale* polysaccharide ameliorates polycystic ovary syndrome via regulating butyrate dependent gut–brain–ovary axis mechanism. Front Endocrinol. (2022) 13:962775. doi: 10.3389/fendo.2022.962775, PMID: 35992123 PMC9389327

[82] Zhang H He Z Chen Y Chao J Cheng X Mao J . Cordyceps polysaccharide improves polycystic ovary syndrome by inhibiting gut-derived LPS/TLR4 pathway to attenuates insulin resistance. Int J Biol Macromol. (2024) 280:135844. doi: 10.1016/j.ijbiomac.2024.135844, PMID: 39326591

[83] Li R Hu R Huang Y Li D Ma X Yang Y. Astragalus polysaccharide alleviates polycystic ovary syndrome by reducing insulin resistance and oxidative stress and increasing the diversity of gut microbiota. Endocrine. (2024) 83:783–97. doi: 10.1007/s12020-023-03553-x, PMID: 37824046

[84] Yong Z Mimi C Yingjie L Yichen G Yansu Y Zhi Z . Mangiferin ameliorates polycystic ovary syndrome in rats by modulating insulin resistance, gut microbiota, and ovarian cell apoptosis. Front Pharmacol. (2024) 15:15. doi: 10.3389/fphar.2024.1457467, PMID: 39376609 PMC11456450

[85] Wang J Jia R Celi P Zhuo Y Ding X Zeng Q . Resveratrol alleviating the ovarian function under oxidative stress by alternating microbiota related tryptophan-kynurenine pathway. Front Immunol. (2022) 13:911381. doi: 10.3389/fimmu.2022.911381, PMID: 35911670 PMC9327787

[86] Dou J Wu Y Hu R Liu J Zhang Y Zhen X . Quinoa ameliorates polycystic ovary syndrome via regulating gut microbiota through PI3K/AKT/mTOR pathway and autophagy. Nutrition Metabolism. (2024) 21:80. doi: 10.1186/s12986-024-00855-3, PMID: 39394588 PMC11468221

[87] Wang T Sha L Li Y Zhu L Wang Z Li K . Dietary α-linolenic acid-rich flaxseed oil exerts beneficial effects on polycystic ovary syndrome through sex steroid hormones—microbiota—inflammation axis in rats. Front Endocrinol. (2020) 11:284. doi: 10.3389/fendo.2020.00284 PMC7326049 32670195

[88] Leung WT Tang Z Feng Y Guan H Huang Z Zhang W. Lower Fiber consumption in women with polycystic ovary syndrome: a Meta-analysis of observational studies. Nutrients. (2022) 14:5285. doi: 10.3390/nu14245285, PMID: 36558444 PMC9785338

[89] Li X Jiang B Gao T Nian Y Bai X Zhong J . Effects of inulin on intestinal flora and metabolism-related indicators in obese polycystic ovary syndrome patients. Eur J Med Res. (2024) 29:443. doi: 10.1186/s40001-024-02034-9, PMID: 39217395 PMC11365155

[90] Jing Xue XL Ping Liu Ke Li Liping Sha Xiaoli Yang Lili Zhu . Inulin and metformin ameliorate polycystic ovary syndrome via anti-inflammation and modulating gut microbiota in mice. Endocr J. (2019) 66:859–70. doi: 10.1507/endocrj.EJ18-0567 31270279

[91] Geng L Yang X Sun J Ran X Zhou D Ye M . Gut microbiota modulation by inulin improves metabolism and ovarian function in polycystic ovary syndrome. Adv Sci. (2025) 12:2412558. doi: 10.1002/advs.202412558, PMID: 40192074 PMC12120758

[92] Li T Zhang Y Song J Chen L Du M Mao X. Yogurt enriched with inulin ameliorated reproductive functions and regulated gut microbiota in Dehydroepiandrosterone-induced polycystic ovary syndrome mice. Nutrients. (2022) 14:279. doi: 10.3390/nu14020279, PMID: 35057459 PMC8781812

[93] Martinez Guevara D Vidal Cañas S Palacios I Gómez A Estrada M Gallego J . Effectiveness of probiotics, prebiotics, and synbiotics in managing insulin resistance and hormonal imbalance in women with polycystic ovary syndrome (PCOS): a systematic review of randomized clinical trials. Nutrients. (2024) 16:3916. doi: 10.3390/nu16223916, PMID: 39599701 PMC11597640

[94] Dong Y Yang S Zhang S Zhao Y Li X Han M . Modulatory impact of *Bifidobacterium longum* subsp. *longum* BL21 on the gut–brain–ovary axis in polycystic ovary syndrome: insights into metabolic regulation, inflammation mitigation, and neuroprotection. mSphere. (2025) 10:e00887-24. doi: 10.1128/msphere.00887-24, PMID: 39898662 PMC11853005

[95] Corrie L Awasthi A Kaur J Vishwas S Gulati M Kaur IP . Interplay of gut microbiota in polycystic ovarian syndrome: role of gut microbiota, mechanistic pathways and potential treatment strategies. Pharmaceuticals. (2023) 16:197. doi: 10.3390/ph16020197, PMID: 37259345 PMC9967581

[96] Zhang F Ma T Tong X Liu Y Cui P Xu X . Electroacupuncture improves metabolic and ovarian function in a rat model of polycystic ovary syndrome by decreasing white adipose tissue, increasing brown adipose tissue, and modulating the gut microbiota. Acupuncture Med. (2022) 40:347–59. doi: 10.1177/09645284211056663, PMID: 34892981

[97] Wu T Xu G Hong X Fan H Zeng J Liu Y . Acupuncture for hormonal readiness and gut microbiota in obese polycystic ovary syndrome: an open-label, randomized controlled trial. Front Endocrinol. (2024) 15:15. doi: 10.3389/fendo.2024.1509152, PMID: 39749020 PMC11693447

[98] Wen Q Hu M Lai M Li J Hu Z Quan K . Effect of acupuncture and metformin on insulin sensitivity in women with polycystic ovary syndrome and insulin resistance: a three-armed randomized controlled trial. Hum Reprod. (2022) 37:542–52. doi: 10.1093/humrep/deab272, PMID: 34907435 PMC8888993

